# PROLIDASE: A Review from Discovery to its Role in Health and Disease

**DOI:** 10.3389/fmolb.2021.723003

**Published:** 2021-08-31

**Authors:** Ireti Eni-Aganga, Zeljka Miletic Lanaghan, Muthukumar Balasubramaniam, Chandravanu Dash, Jui Pandhare

**Affiliations:** ^1^ Center for AIDS Health Disparities Research, Nashville, TN, United States; ^2^ School of Graduate Studies and Research, Nashville, TN, United States; ^3^ Department of Microbiology, Immunology and Physiology, Nashville, TN, United States; ^4^ Pharmacology Graduate Program, Vanderbilt University, Nashville, TN, United States; ^5^ Department of Biochemistry, Cancer Biology, Pharmacology and Neuroscience, Meharry Medical College, Nashville, TN, United States

**Keywords:** prolidase, collagen, wound healing, prolidase deficiency, regulation

## Abstract

Prolidase (peptidase D), encoded by the *PEPD* gene, is a ubiquitously expressed cytosolic metalloproteinase, the only enzyme capable of cleaving imidodipeptides containing C-terminal proline or hydroxyproline. Prolidase catalyzes the rate-limiting step during collagen recycling and is essential in protein metabolism, collagen turnover, and matrix remodeling. Prolidase, therefore plays a crucial role in several physiological processes such as wound healing, inflammation, angiogenesis, cell proliferation, and carcinogenesis. Accordingly, mutations leading to loss of prolidase catalytic activity result in prolidase deficiency a rare autosomal recessive metabolic disorder characterized by defective wound healing. In addition, alterations in prolidase enzyme activity have been documented in numerous pathological conditions, making prolidase a useful biochemical marker to measure disease severity. Furthermore, recent studies underscore the importance of a non-enzymatic role of prolidase in cell regulation and infectious disease. This review aims to provide comprehensive information on prolidase, from its discovery to its role in health and disease, while addressing the current knowledge gaps.

## Introduction

**FIGURE 1:**
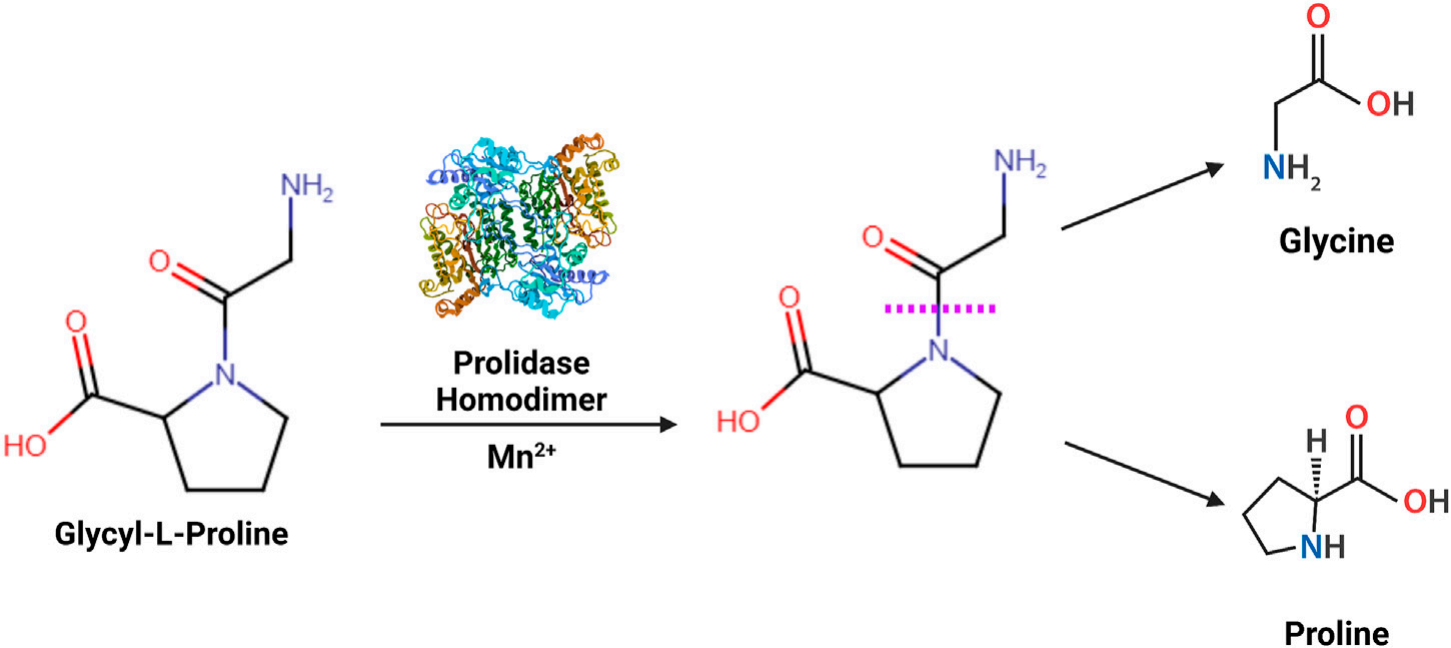
Enzymatic reaction catalyzed by prolidase- Prolidase is a metallopeptidase that functions as a homodimer catalyzing the hydrolysis of imidodipeptides containing a C-terminal proline or hydroxyproline residue (Xaa-Pro). The scissile amide linkage hydrolyzed by prolidase is shown with a purple dashed line.

Prolidase is the only dipeptidase known to catalyze the hydrolysis of a dipeptide containing a C-terminal proline or hydroxyproline into constituent amino acids (Endo et al., 1989; Kitchener and Grunden 2012) (Figure 1). It has many aliases, including peptidase D (PEPD), Xaa-Pro dipeptidase, X-Pro dipeptidase, proline dipeptidase, or imidodipeptidase. Prolidase is present in all three domains of life - archaea, bacteria, and eukaryotes. The enzymatic activity of prolidase releases proline or hydroxyproline in the final stages of the catabolism of endogenous and dietary proteins. The most studied substrate of prolidase are imidodipeptides that are generated during breakdown of collagen - the predominant component of the extracellular matrix (ECM) (Kitchener and Grunden 2012). Collagen is the most abundant structural protein in the human body, attributing to approximately 33% of total protein mass. Proline and hydroxyproline constitute over 20% of collagen (Ramshaw et al., 1998); therefore, collagen degradation generates a considerable amount of dipeptides containing proline or hydroxyproline (Laurent, 1987; McAnulty and Laurent 1987). Prolidase plays an essential role in ECM remodeling by facilitating the intracellular digestion of these dipeptides and promoting the recycling of proline for collagen synthesis (Duong et al., 2006; Surazynski et al., 2008b). Thus, prolidase is required in several physiological and pathological processes such as wound healing, inflammation, angiogenesis, cell proliferation, and carcinogenesis. In this review, we start with a historical snapshot of the discovery of prolidase, then provide an overview of the current body of research, followed by a discussion of the existing knowledge gaps in prolidase research, and conclude with future prospective.

## Discovery of Prolidase

The term “Prolidase” was first coined in 1937 (Grassmann et al., 1932; Bergmann and Fruton 1937; Smith and Bergmann 1979). Researchers, while studying digestion in the intestinal tract, found that crude preparations of erepsin (protein fractions found in the intestinal fluid) had the ability to cleave dipeptides containing a C-terminus proline (glycyl-proline (gly-pro) and alanyl-proline (ala-pro)). It was unclear whether it was a new activity of aminopeptidase or an unknown enzyme. Aminopeptidases can act only on peptide linkages with a hydrogen, while proline with its α-imino group forms special peptide linkages that lack a peptide hydrogen. Thus, the possibility of the enzyme being an aminopeptidase was ruled out. Moreover, upon further study, the enzyme was found to lack the ability to cleave carbobenzoxyglycyl-L-proline (Cbz-pro) and glycylsarcosine. Simultaneously, phenylhydrazine did not inhibit the activity, confirming that this enzyme did not require a peptide hydrogen for action but adapted to cleave linkages containing a proline nitrogen (Bergmann and Fruton 1937). Therefore, the enzyme was given the name “Prolidase,” specifically to classify it as the enzyme that cleaves proline on the C-terminus of a proline containing dipeptide (Grassmann et al., 1932) (Figure 1) and to differentiate it from another enzyme involved in proline metabolism known as prolinase, which cleaves proline on the N-terminus of a proline containing dipeptide (prolyl-glycine). Prolidase was first discovered to cleave gly-pro, but later found to also cleave glycyl-hydroxyproline (Bergmann and Fruton, 1937). C-terminal prolines are not exclusive to dipeptides and may also occur in tripeptides during intestinal digestion. Prolidase activity was narrowed down to dipeptides since the catalytic activity was undetectable with tripeptides and was classified as an imidodipeptidase (Adams and Smith, 1952). Prolidase activation requires manganese and is thus identified as a metal-activated peptidase (Smith and Bergmann, 1979). It requires four manganese ions, but is also functional with other divalent cations like zinc and cobalt, albeit to a lesser degree (Besio et al., 2010; Besio et al., 2013), on the other hand prolidase activity is inhibited by nickel (Miltyk et al., 2008). Prolidase enzymatic function and cofactor requirements are further explored later in this review.

The discovery of prolidase in the intestinal mucosa is significant because prolidase is required for the complete breakdown of proteins such as collagen and gelatin that contain imino-group peptide linkages. Although prolidase’s discovery in intestinal mucosa and its imidodipeptidase activity contributed to increasing knowledge of intestinal fluid composition and protein metabolism, questions arose about prolidase expression in other tissues or cell types. Prolidase was found in human erythrocytes, human kidneys, and swine kidneys (Davis and Smith, 1957). Among the cell types, erythrocytes of both human and horse use manganese as a cofactor and do not differ in substrate specificity (Adams, McFadden et al., 1952; Adams and Smith, 1952). In horse erythrocytes, manganese increases prolidase activity by 26-fold while pyrophosphate, ethylenediaminetetraacetate, fluoride, and citrate were found to inhibit prolidase activity (Adams and Smith, 1952).

## Genetics of Prolidase

**FIGURE 2:**
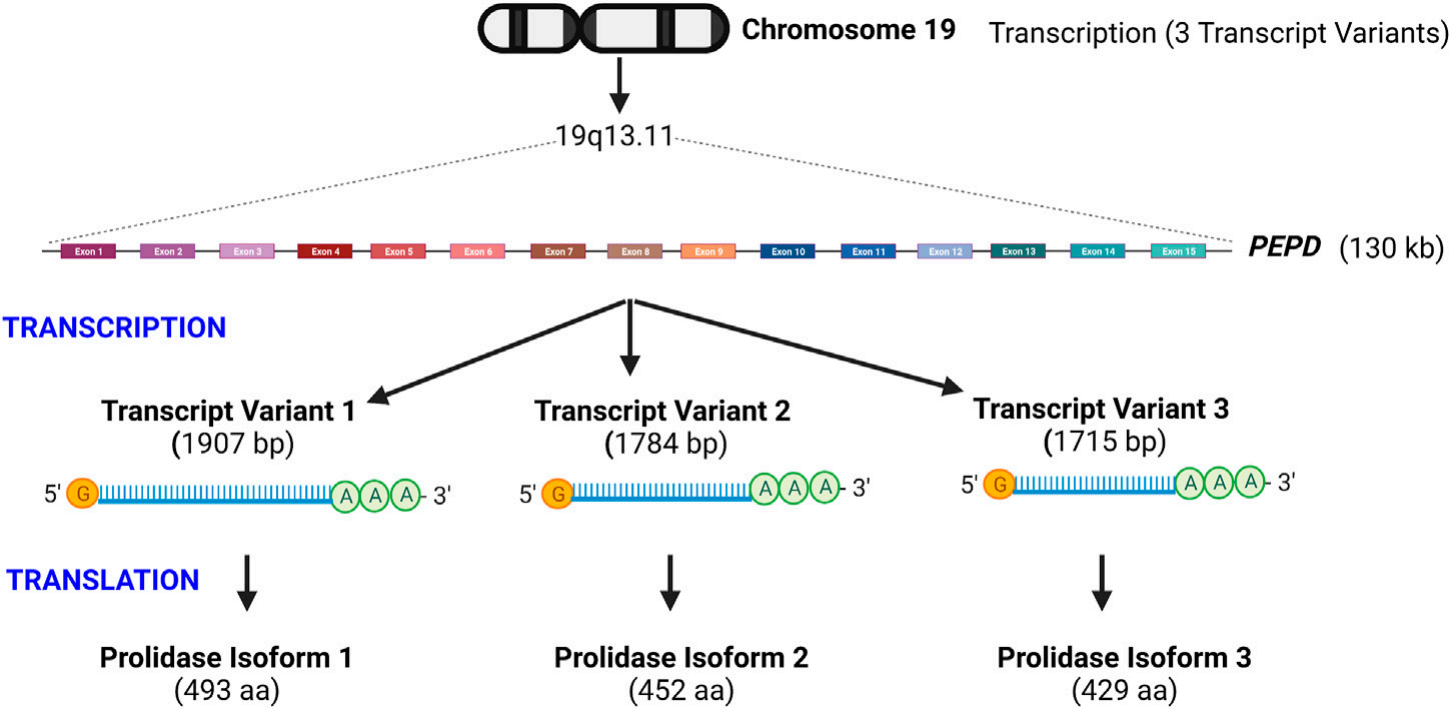
Schematic illustration of prolidase gene organization- Prolidase encoded by the *PEPD* gene is located on chromosome 19q13.11. *PEPD* gene is 130 kb long and encodes three transcript variants that yield three protein isoforms. Isoform 1 is the longest and predominantly studied. Transcript variants 2 and 3 lack two alternate in-frame exons in the central coding region and are translated into proteins of shorter length.

Human prolidase is encoded by the *PEPD* gene, located on the long arm of chromosome 19 at position 13.11 (Gene ID: 5184) (Figure 2). *PEPD* has no TATA box, but S1 nuclease mapping revealed a “CAAT”-like box upstream of the transcription start site, as well as sequences that resemble the Sp1 transcription factor binding sites in this region. (Tanoue et al., 1990a). The 130 kilobases long gene contains 15 exons encoding three mRNA transcript variants (Figure 2). Translation of transcript variant 1 — the longest isoform—yields the canonical 493 amino acid protein. In contrast, the transcript variants 2 and 3 lack two alternate in-frame exons in the central coding region and are translated into 452 and 429 amino acid proteins, respectively (https://www.ncbi.nlm.nih.gov/gene/5184). It is currently unclear whether the three isoforms vary in terms of subcellular localization or functional characteristics. *PEPD* is found in mice, chimps, rhesus monkeys, dogs, cows, rats, chickens, frogs, zebrafish, fruit flies, mosquitos, *Caenorhabditis elegans*, and *Arabidopsis thaliana* (https://www.ncbi.nlm.nih.gov/gene/5184/). The mouse *PEPD* located on chromosome 7 containing 15 exons encodes a 493 amino acid protein. Mouse prolidase is 83.2% homologous at the nucleotide level and 87.2% homologous at the amino acid level to human prolidase (Ishii et al., 1996).

## Prolidase Structure and Activity

Human prolidase protein has been shown to exists in two isoforms; I and II (Uramatsu et al., 2009). Prolidase I (56 kDa), the most common, has a higher affinity for gly-pro and ala-pro dipeptides. In comparison, prolidase II (95 kDa) has a higher affinity for met-pro and a very low affinity for gly-pro (Ohhashi et al., 1988a) (Oono et al., 1990) (Uramatsu et al., 2009). In addition to manganese, calcium can act as a cofactor for prolidase I and cobalt for prolidase II (Ohhashi et al., 1988b). Though the two isoforms are reported, prolidase I is most abundant in human plasma with little to no prolidase II (Cosson et al., 1992). The function of prolidase II is unclear; however, research indicates that patients who are deficient in prolidase have prolidase I deficiency rather than a prolidase II deficiency since in these patients the ability to degrade gly-pro dipeptides is compromised while other C-terminus proline containing dipeptides are degraded (Uramatsu et al., 2009).

Prolidase is a homodimeric enzyme. Hydrophobic and hydrophilic residues are distributed evenly throughout the protein’s amino acid composition (Endo et al., 1989; Tanoue et al., 1990b). Sequence and structure analysis of prolidase shows that it shares similar binding motifs with methionine aminopeptidase, aminopeptidase P (APPro), and creatinase (Bazan et al., 1994). Phenylglyoxal or 1-cyclohexyl-3-(2-morpholinoethyl) carbodiimide metho-p-toluenesulfonate inactivate prolidase while protection is conferred by competitive inhibitor N-acetylcholine, suggesting aspartic and glutamic acid are in the active site (Mock and Zhuang, 1991). Magnesium renders prolidase inactive while occupying the same position as manganese in crystal structures (Wilk et al., 2017). The first structure of prolidase was deduced from the hyperthermophilic archaeon *Pyrococcus furiosus*. The enzyme’s folding pattern was similar to that of APPro and creatinase, confirming previous similarities between these enzymes (Maher et al., 2004). The crystal structure of human prolidase has been determined (Mueller et al., 2006) and is available in the protein data bank (PDB).

The homodimer of human prolidase is oriented in a C2 symmetric pattern. The N-terminal domain of each subunit comprises of amino acid residues 1–184, while the C-terminal domain, which houses the enzyme’s active site, comprises of amino acid residues 185–493. The C-terminal domain contains the “pita-bread fold” found in methionine aminopeptidase, APPro, and creatinase (Maher et al., 2004). Enzymes possessing a pita-bread fold carry a canonical bimetallic center at the bottom of their active site cleft formed by two structurally similar domains of two α-helices and an antiparallel β-sheet. An intermolecular disulfide bond was observed between residues Cys58A and Cys158B, linking two monomers within one dimer. Manganese is the preferred co-factor for prolidase, and the crystal structure suggests that one Mn2+ ion is more tightly bound adjacent to His370, while another is weakly bound adjacent to Asp276 in each subunit. The bridging moiety between the two manganese is essential for catalysis due to the distance, and it is most likely a hydroxide ion instead of a water molecule (Mueller et al., 2006).

Prolidase activity begins with the abstraction of a proton from the bridge water molecule by Glu412 (Wilk et al., 2017). The substrate (gly-pro) diffuses into the active site, leading to a conformational change from an open to a closed state. The Gly-N atom of the substrate interacts with one of the manganese ions, while the Gly-O of the scissile peptide bond interacts with the second manganese ion. His255 is essential for substrate binding, and His377 stabilizes the transitional state. A network of hydrogen bonds from His377, Arg398, His255 holds Gly-Pro to the protein’s active site. His255 binds to the carboxylate group of the substrate proline. The hydroxide ion is held by the manganese ions and the nitrogen on the proline. Manganese ions are stabilized by Asp287, Asp276, Glu452, Glu412, His370, along with the amino group of glycine. The polarization builds a partial positive charge on the carbonyl C-atom of the scissile bond. A nucleophilic attack occurs on the hydroxide ion onto the carbonyl C-atom forming a tetrahedral intermediate. The proton’s shift from the hydroxide to the Pro-N atom coincides with the breaking of the peptide bond. The first product, glycine, leaves the active site while the protein is still in a closed conformation. Another conformation change returns the protein to the open state, and His255 and Trp107 release the proline (Wilk et al., 2017). Other pita-bread enzymes have similar activity and homologs, solidifying the evolutionary relationship (Bazan et al., 1994; Maher et al., 2004).

## Collagen, Prolidase and Wound Healing

Collagen is the most common structural protein in the human body, supporting connective tissues such as skin, bones, cartilage, and ligaments (Laurent, 1987). It accounts for approximately 33% of total protein mass and the predominant component of the extracellular matrix (ECM). Roughly 22% of amino acid residues in collagen strands are either proline or hydroxyproline (Ramshaw et al., 1998). Matrix metalloproteinases (MMPs) are endopeptidases involved in collagen metabolism (Laronha and Caldeira, 2020). The sequential cleavage by multiple MMPs degrades collagens into dipeptides containing proline/hydroxyproline at the C-terminal end that serve as substrates for prolidase (Verma and Hansch, 2007; Yu et al., 2012). Accordingly, the completion of ECM-associated collagen degradation is dependent on the catalytic activity of prolidase. Prolidase catalyzes the final and rate-limiting step in the degradation of collagen, releasing free proline/hydroxyproline, which can recycle into collagen synthesis during matrix remodeling (Surazynski et al., 2008a). Synthesis of collagen is dependent on the availability of proline, which is primarily provided by prolidase, *via* hydrolysis of imidodipeptides generated during collagen breakdown (Albaugh et al., 2017) (Karna et al., 2020). Alternately, proline can be synthesized from glutamine and glutamate or from arginine *via* its conversion to ornithine (Phang et al., 2010). Interestingly, studies have shown that, dietary supplementation of proline does not increase collagen synthesis nor do supplementation of glutamine or glutamate. However, dietary supplementation of arginine and ornithine have been shown to be most effective in increasing collagen deposition (Barbul, 2008). Thus, the homeostasis of collagen synthesis is dependent on the availability of proline *via* cellular metabolism and collagen breakdown generating imidodipeptides. Due to its involvement in collagen remodeling, fluctuations in prolidase activity may therefore, indicate dysfunction in collagen metabolism, altered disease states, or homeostatic imbalance.

Wound healing is a complex and highly regulated process in which different cellular types participate to rapidly and efficiently reconstitute tissue integrity (Gonzalez et al., 2016). It involves intricate and interconnected processes, which occur in four phases: hemostasis, inflammation, proliferation, and remodeling. During hemostasis, damaged tissue triggers the formation of a blood clot. Platelets release growth factors to stimulate the propagation of neutrophils and macrophages, which remove necrotic tissue, debris, and bacteria from the wound. Macrophages release various growth factors and cytokines required for recruiting fibroblasts during the inflammatory phase, such as transforming growth factor-beta (TGFβ). Fibroblasts, the most common cell type of connective tissues, are the primary cells of the proliferative phase that then initiate angiogenesis and collagen formation (Gonzalez et al., 2016; DesJardins-Park et al., 2018). Fibroblasts play a crucial part in synthesizing ECM components, mainly collagen, which provides structure to the wound and replaces the fibronectin–fibrin matrix from the blood clot formed during hemostasis. Collagen turnover provides tensile strength to wounds during the acute and late stages of wound healing. During the early stages of wound healing, fibroblasts actively produce type III collagen. During the remodeling phase, type III collagen is replaced by type I collagen to restore the normal dermal collagen composition (Xue and Jackson, 2015). Collagen turnover drives the remodeling of the ECM and is critical for effective wound healing. Wound repair depends upon a supply of proline *via* collagen recycling for the healing process. The recycling and remodeling of collagen is therefore dependent on prolidase activity to make free proline available from collagen degradation for resynthesis. This was demonstrated in rats where during wound healing, prolidase activity increased in rats with burned skin compared to rats without burns (Umemura et al., 1980). Prolidase activity has been reported to be higher in wound fluid from patients with chronic pressure wounds, and prolidase mRNA levels were found to be higher in scar tissue fibroblasts, keratinocytes, and endothelial cells (Senboshi et al., 1996; Oono et al., 1997). Also, prolidase gene expression is upregulated in old scar tissue compared to uninjured skin (Senboshi et al., 1996). On the other hand, research on aged fibroblasts shows a decrease in collagen, along with a decrease in prolidase activity (Pałka et al., 1996). Over time, fibroblasts in the dermis become senescent, and the number of collagen metabolic enzymes, including prolidase, decreases. Because manganese is a co-factor for prolidase, drugs that chelate metal ions, such as daunorubicin, doxycycline, and anthracycline, inhibit prolidase activity and lead to poor wound healing as a side effect (Muszyńska et al., 2000; Karna et al., 2001; Muszyńska et al., 2001). A common symptom and identifier of Prolidase deficiency (PD) is defective wound healing. Proline availability is critical for proper collagen synthesis, and the body maintains a tight balance over proline availability (Albaugh et al., 2017). PD patients suffer from unhealing ulcerative wounds and bone deformities due to the excretion of proline-containing dipeptides due to inactive prolidase.

Diabetes mellitus (DM) may impair wound healing, though the pathophysiology of impaired wound healing in DM is not fully understood (Huang and Kyriakides, 2020). High blood glucose decreases collagen production and fibroblast migration and inhibits inflammatory cell proliferation, leading to chronically non-healing ulcers (Okonkwo and DiPietro, 2017). Serum prolidase levels are elevated in patients with Type 2 diabetes and diabetic foot ulcers (Eren et al., 2013). Lower or defective prolidase is associated with impaired wound healing, suggesting that in the case of DM, other factors may also affect wound healing.

Keloids are an abnormal proliferation of scar tissue that form at sites of a cutaneous wound (Hinz, 2016). Keloids result from inflammation in the dermis, overactivation of myofibroblasts and other connective tissue cells, and excess ECM proteins. This leads to an atypical overgrowth of the scar that extends past the area of the wound. Collagen synthesis, mainly accumulation of type I collagen, is about 20-fold higher in keloid than in healthy skin. Collagen accumulates due to an imbalance in the homeostasis of collagen turnover by fibroblasts, and collagenase activities are also significantly higher in keloid tissues (Hinz, 2016; Karppinen et al., 2019). Prolidase mRNA and protein expression are increased in keloids in comparison to healthy skin. This increase in activity may enhance collagen biosynthesis due to the increase in the proline pool, providing a constant resource that enhances collagen deposition (Duong et al., 2006).

## Prolidase and Cellular Regulation

### Enzyme Activity Dependent Regulation

**FIGURE 3:**
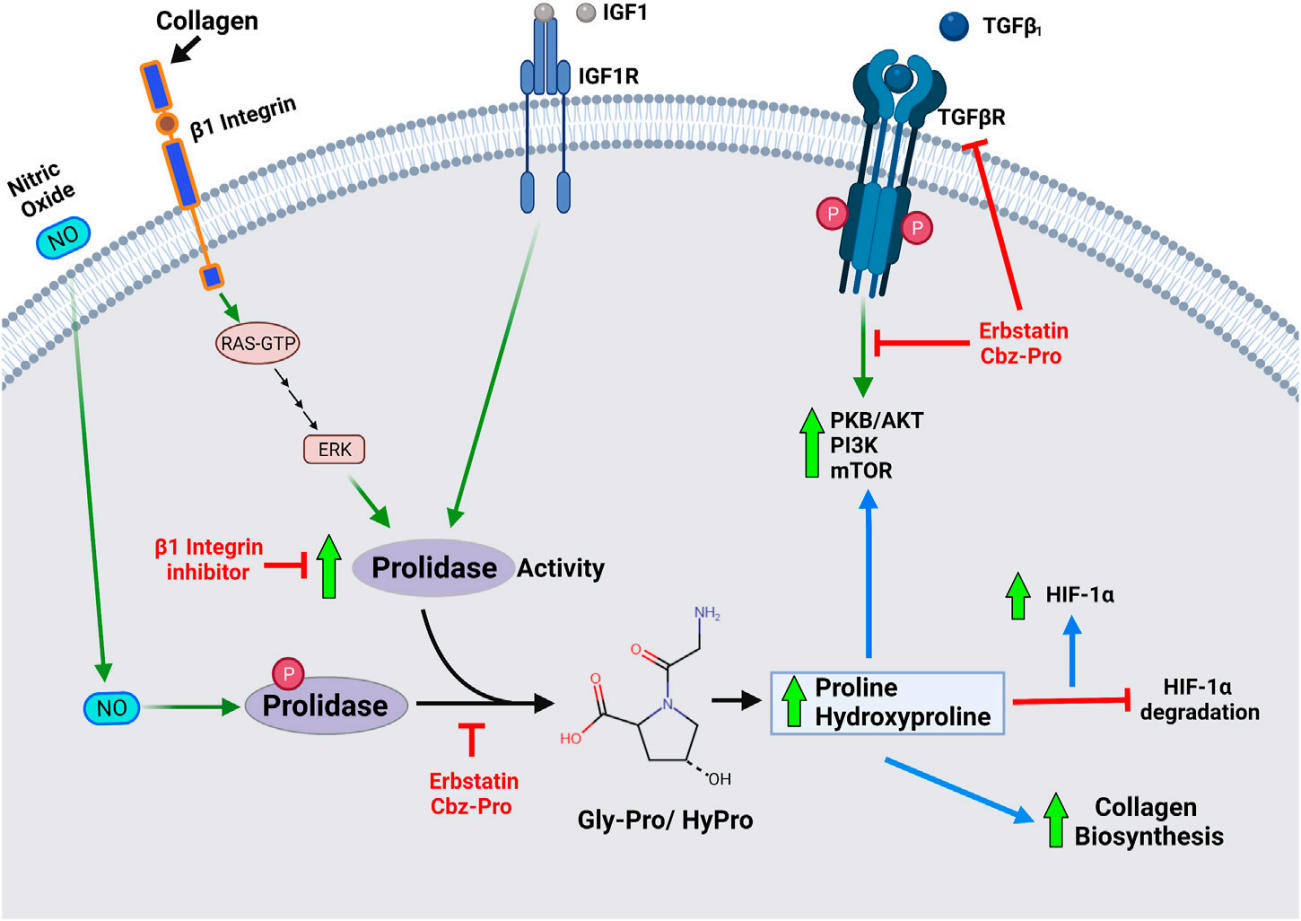
Diagram depicting role of prolidase enzymatic activity in cellular regulation- Prolidase plays a pivotal role in collagen metabolism, matrix remodeling and cell growth. Therefore, prolidase, is essential in several physiological processes such as wound healing, inflammation, angiogenesis, cell proliferation, and carcinogenesis. Accordingly, primary mechanisms that are known to regulate prolidase activity include-activation of the β1 integrin receptor, insulin-like growth factor 1 receptor (IGF-1R), and transforming growth factor (TGF) receptor. Stimulation of these receptors by their ligands-collagen, IGF1 and TGFβ1 respectively affect prolidase enzyme activity and downstream processes associated with cell proliferation and growth. Nitric oxide (NO) mediated phosphorylation of prolidase has been shown to increase enzyme activity, while inhibition of phosphorylation renders a less active prolidase enzyme. The products of prolidase activity-proline and hydroxyproline stimulate collagen biosynthesis, stabilize HIF-1α by inhibiting its degradation and induce TGF receptor mediated pathways.

A plethora of reports on the clinical importance of prolidase, especially in the context of prolidase deficiency, are available. However, studies focused on the regulation of prolidase are limited. As aforementioned, prolidase plays a pivotal role in collagen metabolism, mainly in recycling collagen *via* its degradation and resynthesis. Studies suggest that prolidase activity is regulated by interactions between the ECM and surface receptors because prolidase activity increases with cell growth and density. Thus, it was logical to presume that factors involved in the regulation of collagen may also regulate prolidase activity. The first reference on the regulation of prolidase activity was through the study of collagen and β1 integrin receptor (Palka and Phang, 1997) (Figure 3). Integrin receptors are essential for integrating ECM signals by transmitting signals bidirectionally between the ECM and cytoplasm. They are heterodimeric transmembrane receptors composed of eighteen α subunits and eight β subunits that are non-covalently assembled into at least 24 different functional receptors (Mezu-Ndubuisi and Maheshwari, 2021). The most prominent integrin receptor, β1 integrin, is mainly responsible for attachment to the ECM. Upon stimulation, β1 integrin has been shown to mediate cell motility, survival, proliferation and differentiation. Type I collagen is a ligand for the β1 integrin receptor, and it was shown that the addition of collagen led to an increase in prolidase activity (Palka and Phang, 1997; Zeltz et al., 2014). Subsequent studies demonstrated that β1 integrin triggers the Ras-Raf-MEK-ERK pathway to stimulate prolidase catalytic activity independent of prolidase protein expression. They further demonstrated that inhibitors of β1 integrin inhibit the Ras-Raf-MEK-ERK pathway with a concomitant decrease in prolidase activity, strongly suggesting that prolidase activity is regulated by this pathway (Surazyński et al., 2004). Another example of the factor that stimulates collagen biosynthesis and regulates prolidase activity is the insulin-like growth factor-1 (IGF1) (Figure 3). When IGF1 interacts with its receptor IGF1R, it stimulates collagen biosynthesis, and this interaction results in an increase in prolidase activity in human dermal fibroblasts (Miltyk et al., 1998; Blackstock et al., 2014). Prolidase activity is also influenced by the induction of oxidative stress that affects IGF1 receptor signaling (Sienkiewicz et al., 2004).

Prolidase requires activation for its enzymatic activity. Phosphorylation of prolidase has been identified as a potential mechanism for regulating its enzymatic activity (Figure 3). Prolidase activity was reduced in fibroblasts treated with erbstatin, a naturally occurring inhibitor of tyrosine-specific protein kinase. The same study showed that an anti-phosphotyrosine antibody also reduced prolidase activity in a dose-dependent manner while phosphotyrosine phosphatase 1B increased prolidase activity (Surazyński et al., 2001). Serine/threonine phosphorylation of prolidase was also reported in fibroblasts treated with nitric oxide, which mediates inflammatory responses, wound repair, and angiogenesis (Surazynski et al., 2005). In another study, prolidase activity was shown to be decreased in fibroblasts from fasting rats. Fasting results in ATP depletion, leading to accumulation of inorganic phosphate and pyruvate kinase activity inhibition, affecting phosphoenolpyruvate (PEP) conversion into pyruvate. The authors postulated that the accumulating PEP may form a complex with prolidase and prevent its phosphorylation or stimulate its dephosphorylation; however, the exact mechanism remains unknown (Cechowska-Pasko et al., 2004). The same research group demonstrated that thrombin, the enzyme that converts fibrinogen to fibrin in the blood clotting cascade, inhibits prolidase expression while inducing its phosphorylation in colorectal adenocarcinoma cells (Lundblad et al., 2004; Karna et al., 2012). However, thrombin binds to the β1 integrin receptor and increases prolidase activity in human dermal fibroblasts (Surazyński et al., 2005).

Proline and hydroxyproline, the products of prolidase enzyme activity, have been shown to increase nuclear hypoxia-inducible factor levels (HIF-1α) levels by inhibiting HIF-1α degradation (Surazynski et al., 2008a) (Figure 3). HIF-1α is associated with wound repair as it enhances genes associated with inflammation, angiogenesis, and fibrosis such as vascular endothelial growth factor (VEGF), glucose transporter-1 (GLUT-1), and transforming growth factor-β1 and 2 (TGF-β1 and 2) (Lokmic et al., 2012). HIF-1α is hydroxylated at proline residues by prolyl hydroxylase under normoxic conditions, allowing for HIF-1α ubiquitination by the von Hippel-Lindau (VHL) E3 ubiquitin ligase, leading to proteasomal degradation. In the absence of proteasomal degradation, HIF1α accumulates and translocates into the nucleus to transcribe associated genes. Overexpression of prolidase results in increased HIF-1α levels - leading to elevated HIF-1α-dependent gene products such as VEGF and GLUT-1. This increase in HIF-1α-dependent gene products was attributed to products of prolidase activity. To determine if prolidase was directly involved in the increased production or decreased degradation of HIF-1α, the oxygen-dependent degradation domain (ODD) of HIF-1α was fused to a luciferase gene (Luc-ODD). Overexpression of prolidase increased Luc-ODD levels, suggesting that HIF1 degradation was disrupted. The same effect was also observed with the addition of proline or hydroxyproline in a dose-dependent manner. The authors concluded that prolidase activity, which produces proline and hydroxyproline, inhibits the degradation of HIF-1α *via* the VHL dependent proteasomal pathway (Surazynski et al., 2008a).

TGF-β1 is a cytokine that plays a key role in wound healing, vascular injury, cell proliferation, and differentiation. It has been linked to fibrosis development due to its ability to regulate and stimulate collagen biosynthesis (Kubiczkova et al., 2012). TGF- β1 can cause fibrosis by inducing protease inhibitor production, which prevents the enzymatic breakdown of the ECM, particularly collagen (Kim et al., 2018). Inhibitors of prolidase activity decreased TGF-β1 and its receptor expression in fibroblasts (Surazynski et al., 2010). This results in the downregulation of TGFβ receptor-stimulated kinases such as Protein Kinase B (PKB or AKT), phosphatidylinositide 3-kinases (PI3K), and mammalian target of rapamycin (mTOR). Furthermore, the addition of proline and hydroxyproline reversed the effect, thus leading to the conclusion that the products of prolidase activity, rather than the prolidase protein, affect TGFβ downstream signaling (Figure 3).

Estrogen, the primary female sex hormone, is frequently linked to breast cancer and the female reproductive system. Estrogen affects the balance of collagen biosynthesis and acts as a regulator in collagen metabolism. Estrogen has been linked to an overall increase in collagen deposition in wound healing, as estrogen levels have been shown to decrease with age, resulting in decreased collagen and impaired wound healing (Horng et al., 2017). Estrogen was shown to upregulate prolidase activity with a concomitant increase in collagen biosynthesis in MCF7 breast cancer cells (Miltyk et al., 1999). In the presence of estradiol, prolactin inhibited prolidase activity while estrogen receptor α (ERα) upregulated prolidase activity (Surazynski et al., 2013a; Surazynski et al., 2013b).

Prolidase has been recently shown to play a novel role in exacerbation of HIV-1 infection by cocaine treatment. HIV-1 neuropathogenesis has been reported to worsen in HIV-1 infected patients who abuse drugs like cocaine. HIV-1 is usually unable to cross the blood-brain barrier (BBB). The BBB is a semipermeable and selective barrier that separates the circulating blood from the brain to protect the central nervous system. The BBB consists of the basement membrane, which is primarily composed of type IV collagen. Cocaine has been shown to increase prolidase activity in brain microvascular endothelial cells, causing a weakened basement membrane and increased permeability in HIV-1-infected monocytes. Cocaine-dependent nitric oxide induction was also shown to increase prolidase phosphorylation, resulting in increased activity (Ysrayl et al., 2019).

### Non-enzymatic Activity of Prolidase in Cellular Regulation

**FIGURE 4:**
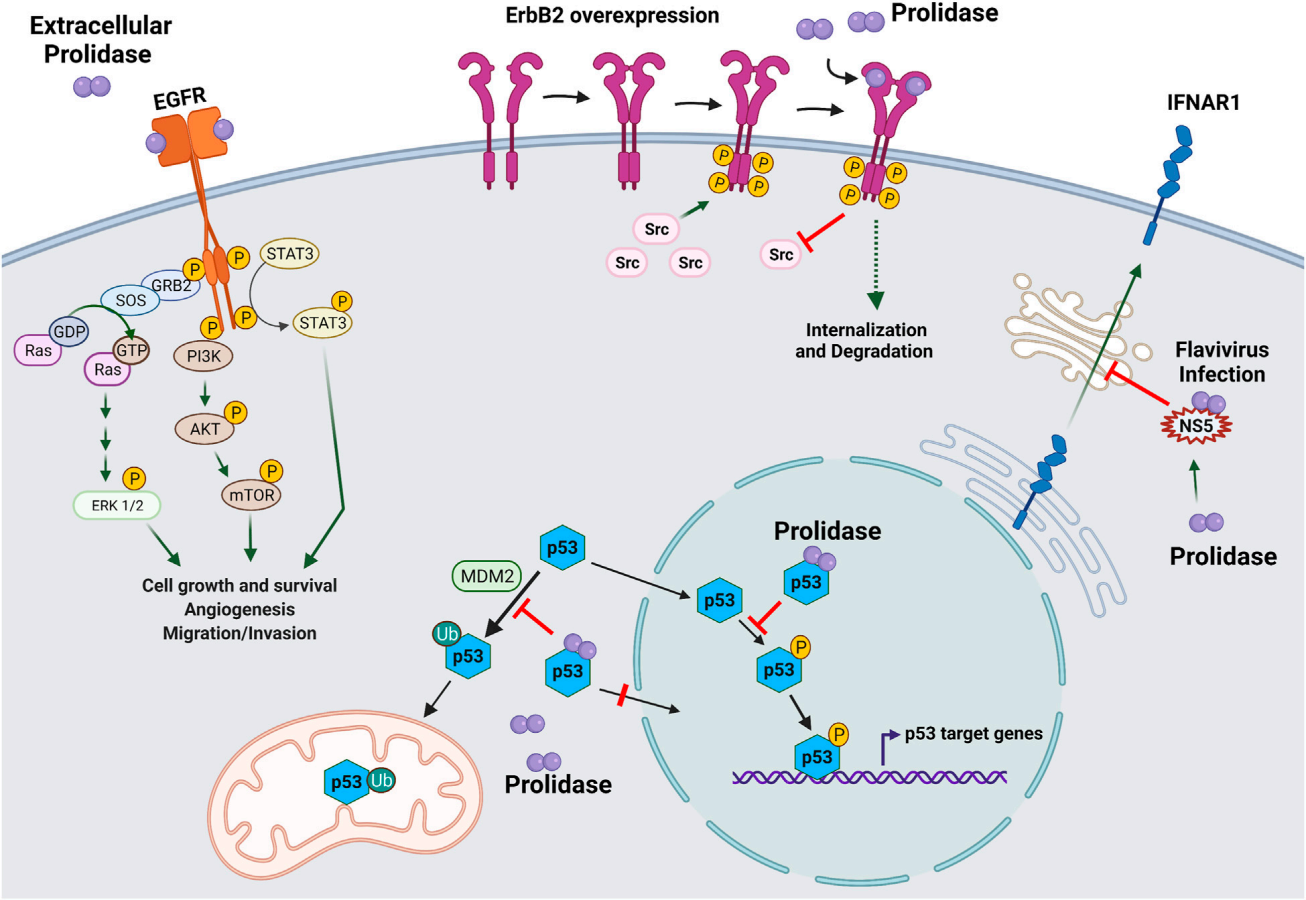
Enzyme activity independent role of prolidase in cellular regulation. A novel role of prolidase as a biological regulatory protein independent of its catalytic activity has recently emerged. Prolidase was demonstrated to function as a ligand for epidermal growth factor receptor (EGFR/ErbB2) and HER2/ErbB2 receptor. Binding of prolidase extracellularly to EGFR receptor stimulated downstream pathways important in cell proliferation and growth such as extracellular signal-regulated kinase (ERK)1/2, phosphoinositide 3-kinase (PI3K), protein kinase B (Akt), mammalian target of rapamycin (mTOR), and signal transducer and activator of transcription 3 (STAT3). Likewise, prolidase was shown to bind overexpressed HER2/ErbB2 receptor and inhibit downstream signaling *via* disruption of its association with Src causing internalization and degradation. Prolidase, independent of its catalytic activity was demonstrated to regulate p53 tumor suppressor protein *via* direct binding. A dual mechanism involving inhibition of p53 phosphorylation and disruption of its interaction with MDM2 resulted in sequestration of p53 in the cytosol thereby preventing both transcription-dependent and transcription-independent activities of p53. Prolidase plays a novel role as a regulator of type I interferon (IFN-I) immune response during flavivirus infection. It is essential for maturation and trafficking of IFN-I receptor (IFNAR1). During infection, the nonstructural protein 5 (NS5) produced by flaviviruses was shown to bind prolidase compromising IFN-1 response.

The majority of research on prolidase focuses on its catalytic activity in collagen metabolism. However, recently enzyme activity independent functions of prolidase have emerged (Figure 4). In a novel finding, prolidase was shown to function as a ligand of EGFR (ErbB1), and its binding stimulated downstream signaling proteins in the EGFR pathway (Yang et al., 2013) (Figure 4). This activation of signaling was not dependent on the catalytic activity of prolidase. Interestingly, prolidase does not have any structural homologies to other EGFR ligands. The binding of prolidase resulted in the phosphorylation of EGFR and the activation of key signaling molecules downstream of EGFR, such as Akt (Protein kinase B), STAT3 (Signal transducer and activator of transcription 3), and ERK (Extracellular signal-regulated kinase). This activation occurred only when prolidase was present extracellularly. This is intriguing because prolidase is generally considered a cytosolic protein and is not known to be secreted in the extracellular milieu even during collagen or ECM remodeling. It is plausible that prolidase is released from damaged cells and tissue, which then binds to EGFR. The binding affinity of prolidase to EGFR was much lower than EGF, the known ligand of EGFR; however, mutated prolidase was shown to silence EGFR signaling (Yang et al., 2016). Follow up studies by the same group also demonstrated that prolidase and an enzymatically inactive mutant function as ligands for the ErbB2 (HER2) receptor (Figure 4). ErbB2 is an oncogenic receptor tyrosine kinase overexpressed in a subset of human breast cancer and other cancers (Joshi and Press, 2018). Recombinant human prolidase binds as a dimer with a high affinity to ErbB2 monomers resulting in their dimerization. Prolidase binding was shown to strongly inhibit ErbB2-overexpressing tumors in mice, whereas it did not impact tumors without ErbB2 overexpression (Yang et al., 2014). In cells overexpressing ErbB2, recombinant prolidase was shown to bind to preformed ErbB2 dimers. The inhibition in tumors due to prolidase binding was attributed to ErbB2 depletion and disruption in oncogenic Src signaling. The binding of prolidase and its enzymatic mutant to the receptor led to potent inhibition of ErbB2 (dimeric form)-overexpressing tumors in mice, and the phenomena was observed only when ErbB2 is overexpression is not present (Yang et al., 2015; Yang et al., 2016).

Another novel non-enzymatic activity of prolidase (and its mutants) is as a regulator of p53 tumor suppressor (Yang et al., 2017). Prolidase was shown to bind to p53 in the nucleus and in the cytoplasm and suppress both transcription-dependent and transcription-independent activities of p53 (Figure 4). Prolidase suppresses p53 from functioning as a transcription factor by sequestering nuclear p53 and inhibiting its phosphorylation. In addition, prolidase acts as a mouse double minute 2 homolog (MDM2) competitor preventing its association with p53, and consequent ubiquitination and translocation to mitochondria. This inhibitory effect is abrogated upon disruption of the association of prolidase with p53 and the ensuing p53 transcription-dependent and independent functions, result in cell death and tumor regression. Interestingly, this is the first report demonstrating the presence of prolidase in the nucleus (Yang et al., 2017).

In addition to the above mentioned non-enzymatic roles of prolidase, a unique role of prolidase in flavivirus infection has been reported (Lubick et al., 2015). During viral infections, a type I interferon (IFN-I) immune response is induced that signals through IFN-I receptor (IFNAR1). To counter this immune response, viruses are known to produce proteins that antagonize the response. For instance, the nonstructural protein 5 (NS5) produced by flaviviruses, acts as an antagonist of IFN-I. Investigation of flavivirus antagonism of IFN-1 response revealed that prolidase is required for IFNAR1 receptor maturation and that flavivirus NS5 downregulates IFNAR1 by sequestering prolidase and thus inhibiting the maturation of IFNAR1 (Lubick et al., 2015) (Figure 4). Improper maturation of IFNAR1 in prolidase deficient patients may contribute to immune issues observed in these patients (Lubick et al., 2015).

## Prolidase Deficiency

Prolidase Deficiency (PD) is a rare autosomal recessive disorder marked by elevated levels of imidodipeptides (proline/hydroxyproline-containing dipeptides excreted in urine) (Cottin et al., 2020). One of the first reports of PD described a patient with an unusual bone disorder characterized by excessive excretion of dipeptides containing proline, implying an increase in bone collagen turnover. Though the patient’s bones were deformed, the skin appeared normal; while it was concluded that there was a defect in collagen synthesis, the causative agent was however, unknown (Seakins, 1963). Another subsequent study, described a patient with skeletal, facial, and cardiovascular abnormalities, as well as high imidodipeptide excretion. The patient excreted increased quantities of bound hydroxyproline, indicating increased collagen turnover or defective catabolism by hydroxyproline oxidase (Goodman et al., 1968). Although the patient’s condition resembled lathyrism, the decreased collagen stability did not fully match the symptoms, implying other unidentified mechanisms. The dipeptides found in urine were extracted and tested *in vitro* as prolidase substrates. The excreted dipeptides were hydrolyzed by exogenous prolidase, leading to the conclusion that the patient had a prolidase function defect due to a deficiency. Additional studies by another research group defined the lathyrism-like symptoms as PD (Buist et al., 1972).

PD was categorized as an autosomal recessive disorder by studying the immediate family tree in a patient with imidopeptiduria (Powell et al., 1974). Prolidase activity was reduced in the patient’s red and white blood cells, in addition to the clinical indication of defective collagen metabolism. White blood cells from the patient’s mother and maternal grandfather also had lower prolidase activity compared to healthy controls. Prolidase enzymatic activity was normal in the maternal grandmother, signifying that prolidase is an autosomal recessive disorder (Powell et al., 1974). Both hydroxyproline and proline were found on the C-terminus of excreted dipeptides, and excreted hydroxyproline level was ten times higher than that of healthy people. Prolinase levels remained normal, bolstering the theory that the dipeptides were excreted due to a prolidase deficiency. This was the first time that the symptoms of prolidase deficiency were linked to genetics.

The efficiency of recycling proline is approximately 90–95% in healthy individuals (Jackson and Heininger, 1975). Prolidase is essential in collagen turnover, and synthesis, and defects in prolidase lead to blockage in regular recycling of collagen because of a decreased pool of available proline (Spodenkiewicz et al., 2020). Fibroblasts are primarily known for leading collagen metabolism and have high levels of prolidase activity. Disturbances in prolidase activity in fibroblasts are associated with connective tissue abnormalities like hypermobile joints. Proline deprivation due to reduced prolidase activity may cause improper wound healing (Dolenga and Hechtman, 1992). Prolidase is necessary for cell survival, and cultured fibroblasts from PD patients exhibited necrosis-like cell death due to increase in undigested prolidase substrates (Forlino et al., 2002). Collagen metabolism is disrupted in all PD patients, affecting bone structure and wound healing. Improperly formed bone structure causes dysmorphic facial features such as a low anterior and posterior hairline, widely spaced/bulging eyes, a depressed nasal bridge, thin upper lip vermilion, and prognathism (Falik-Zaccai et al., 2010). Due to the importance of collagen turnover during wound healing events, most PD patients have large ulcerative wounds that are unable to heal correctly (Goodman et al., 1968; Dunn et al., 2011; Süßmuth et al., 2020). Collagen constituents are excreted rather than recycled, which may impede wound healing in PD patients. Various other symptoms are associated with PD including dermatological lesions, developmental delay, splenomegaly, repetitive infections, and autoimmune manifestations (Nir et al., 2016; Spodenkiewicz et al., 2020; Kenig et al., 2021). In addition to chronic and recurrent ulcers, other skin manifestations including dermatitis, erythematous papular eruptions, impetigo-like eruptions, pseudo-psoriasis skin lesions, and pruritic eczematous lesions have been reported (Falik-Zaccai et al., 2010; Spodenkiewicz et al., 2020).

Another common symptom is breathing problems stemming from a cystic fibrosis (CF)-like phenotype (Luder et al., 2007; Cottin et al., 2020). Immunoglobulin levels are higher in PD patients due to the binding of gamma globulins to prolidase substrates in blood serum, and PD patients suffer from recurrent infections due to open sores on the skin and infections in the respiratory tract (Jackson and Heininger, 1975; Sheffield et al., 1977; Cleary et al., 1994). Other than collagen, these imidodipeptide substrates are common in immunoglobulins and Clq in complement (Reid, 2018). Another immune related symptom includes a systemic lupus erythematosus (SLE)-like phenotype (Butbul Aviel et al., 2012; Kurien et al., 2013; Chidambaram et al., 2021). The mechanism by which SLE is associated with PD is not clear although, as in the case of CF, a role for a high proline containing defective C1q complement has been proposed (Chidambaram et al., 2021).

Numerous clinical studies on PD have observed intellectual disabilities and developmental delays in patients. The deficiency of prolidase in mice also shows intellectual delays and disabilities (Besio et al., 2015) which have now become associated with PD, implicating the importance of prolidase in brain health and function. However, the role of prolidase in brain health is understudied. A study suggested that the presence of iminodipeptides leads to weakened cerebral vessels inducing superoxide production (Yasuda et al., 1999). Prolidase and other mental disorders are discussed later in this review. Though there are multiple phenotypes associated with PD, not all exhibit the same severity amongst all patients and even patients let alone in patients belonging to the same family tree.

### Genetics of Prolidase Deficiency

Studies of prolidase activity in healthy patients’ sera revealed that a dysfunctional enzyme more likely causes PD rather than a reduction in the amount of enzyme available. Manganese is required for prolidase activity, and the addition of manganese did not activate prolidase in PD patients suggesting that an inactive enzyme may be due to mutations in the *PEPD* gene (Ohhashi et al., 1988a). Amplifying the coding region of prolidase from PD patients revealed a G to A substitution in position 826 in exon 12, resulting in an aspartic acid instead of asparagine and consequently producing a defective enzyme. Gene expression studies in NIH3T3 cells confirmed that, unlike the enzymatically active wild-type prolidase, the mutant prolidase lacked activity thus establishing the importance of G to A substitution in PD. This discovery, at the time, meant gene replacement therapy might be possible (Tanoue et al., 1990a). However, in addition to the G to A substitution, the absence of the 14th exon of *PEPD* gene was reported to result in prolidase deficiency in another PD patient, (Tanoue et al., 1990b).

Based on previous studies utilizing a monoclonal antibody specific to wild-type human prolidase, it was suggested that PD was due to a missing portion of the enzyme since the antibody was unable to detect the enzyme in patients (Endo et al., 1987). A single nucleotide base substitution of C to T at position 551 in exon 8 of *PEPD* gene was identified in a PD patient, that yielded a stop codon instead of arginine, resulting in a 20 kDa defective prolidase (Kikuchi et al., 2000). Recently, a single nucleotide deletion, causing a frameshift mutation and encoding a premature stop codon in the *PEPD* gene was reported to disrupt the enzymatic function of prolidase (Kiratli Nalbant et al., 2019). Likewise, another study reported a homozygous nonsense variation in exon 5 of the *PEPD* gene that resulted in a stop codon and premature truncation of the protein at codon 140 resulting in PD (Chidambaram et al., 2021). Overall, 35 *PEPD* variants have been identified. There are 16 missense/nonsense variants, nine splice variants, nine insertions/deletions, and one large deletion. The majority of missense mutations are found in the catalytic domain, located in exon 12 (six mutations). A large number of mutations are also found in exons 8 (six mutations) and 14 (four mutations), all of which are in the C-terminus. Due to the wide range of phenotypes, the extent of prolidase inactivation varies. Disruption of the catalytic center, displacement of important active site residues, and the rigidity of the open-closed conformation required for substrate uptake and release are likely mechanisms for inactivation, that are based on the location of the mutations (Spodenkiewicz et al., 2020).

### Diagnosis of Prolidase Deficiency

To date, different methods have been developed for diagnosing prolidase deficiency. They are mainly based *1*) on the determination of enzyme activity in erythrocytes, leukocytes, and/or skin fibroblast cultures of PD patients or *2*) on the screening of X-Pro imidodipeptides massively excreted in their urine. The oldest method for detecting prolidase activity utilizes Chinard’s reagent, which relies on a colorimetric reaction of proline upon hydrolysis of the X-Pro peptide substrate (Myara et al., 1982). Other methods to determine prolidase activity and imidodipeptides include amino acid analysis, HPLC-API/MS, and MALDI-TOF (Viglio et al., 2006). The preferred method of establishing PD diagnosis is sequence analysis of *PEPD*, followed by gene-targeted deletion/duplication analysis if only one or no pathogenic variant is found (Lupi et al., 2008). Alternately, in specific populations, targeted analysis for pathogenic variants such as Arg265Ter in exon 11 and Ser202Phe in exon 8 is performed first followed by sequence analysis (Ferreira and Wang, 1993; Spodenkiewicz et al., 2020).

### Therapeutics for Prolidase Deficiency

Prolidase deficiency being a rare disorder, not many therapeutics are currently available. Gene therapy is a viable option but has not been accomplished so far. The clinical features of PD are characterized by chronic recurrent ulcers, especially on the legs; therefore, treatment with topical ointments has been suggested as a possible therapy. Ointments containing glycine and proline as well as growth hormone (GH) have been used since prolidase deficiency is associated with GH deficiency (Arata et al., 1986; Monafo et al., 2000). A few therapies have tried to alleviate symptoms by replacing deficient enzyme in PD patients (Viglio et al., 2006). This included the use of manganese-activated red blood cells from healthy patients to overcome prolidase deficiency; however, they have been largely unsuccessful (Hechtman et al., 1988). Another approach was adenovirus-mediated gene therapy using human prolidase cDNA transferred into PD fibroblasts from patients. Prolidase activity increased up to approximately 7.5 times in PD fibroblasts compared to normal fibroblasts (Ikeda et al., 1997). Other approaches include targeted delivery of liposome-encapsulated prolidase to PD patients’ fibroblasts (Perugini et al., 2005). Unfortunately, these proposed therapies for PD are understudied, short-lived, inaccessible, and have a low success rate.

## Prolidase and Other Diseases

The majority of research on prolidase focuses on PD and defective wound healing, however, perturbations in prolidase activity have been reported in various other disorders and diseases including cancer, diabetes, cardiovascular disease and others. These alterations in prolidase activity are mainly associated in pathological conditions with enhanced collagen breakdown or turnover, while in some conditions the cause of the observed variations in activity remains to be elucidated. Prolidase activity has been shown to be directly associated with oxidative stress and suggested to be a useful biochemical marker of oxidative stress in various disorders (Yildiz et al., 2008; Gecit et al., 2012; Kokacya et al., 2014; Ozbay et al., 2020). Oxidative stress is described as an imbalance between reactive oxygen species (ROS) and antioxidants due to overproduction of ROS (Pizzino et al., 2017). Multiple studies provide evidence that oxidative stress stimulates collagen breakdown *via* inductions of MMPs and inducing an inflammatory microenvironment (Kim et al., 2016). These ROS-induced alterations of the ECM microenvironment have been suggested to be responsible for modulating prolidase activity by making available the substrate *via* breakdown of collagen.

### Prolidase and Liver Diseases

Excessive accumulation of collagen and other matrix components causes liver fibrosis, which can progress to liver cirrhosis in most chronic liver diseases. Chronic hepatitis C virus (HCV) infection, alcohol abuse, and non-alcoholic steatohepatitis (NASH) are some of the most common causes of liver fibrosis (Bataller and Brenner, 2005). Myara et al. discovered a link between serum prolidase activity (SPA) and chronic liver diseases, suggesting that plasma prolidase activity monitoring could help assess fibrotic processes in chronic liver disease (Myara et al., 1984). Patients with chronic liver diseases had higher SPA than healthy individuals; however, only a few biopsy-positive cirrhosis patients had increased SPA. A study using a rat model of cirrhotic liver fibrosis suggested that SPA levels may be higher in the early stages of fibrosis and then drop as the disease progresses. This increase in circulating prolidase was not due to leakage as there was no correlation with alanine aminotransferase, an enzyme that leaks into the bloodstream following liver damage (Abraham et al., 2000). Prolidase may increase in the first stages of fibrosis due to the sudden accumulation of collagen and falls at later stages since the liver may not sustain prolidase production.

Brosset et al. investigated prolidase enzyme activity in patients with alcoholic liver disease (Brosset et al., 1988). Patients with alcoholic hepatitis have been shown to have significantly higher prolidase activity in their plasma than patients with stable cirrhosis. NASH is a form of advanced non-alcoholic fatty liver disease (NAFLD) due to buildup of fat in the liver, causing inflammation and liver damage. NASH also leads to fibrosis before cirrhosis. The serum prolidase levels have been used as a non-invasive indicator of the disease’s progression, distinguishing between simple steatosis and non-alcoholic steatohepatitis (Kayadibi et al., 2009; Arora and Sharma, 2012). Another study revealed that liver biopsy specimens from patients with NASH had significantly higher serum prolidase enzyme activity than healthy controls, (Horoz et al., 2010).

HCV is a single-stranded enveloped positive-sense RNA virus belonging to the Flaviviridae family. It infects the liver, leading to inflammation and fibrosis. It also causes cirrhosis, steatosis, and hepatocellular carcinoma (Ray and Thomas, 2014). Studies suggest that structural and non-structural HCV proteins may directly trigger stellate cell (liver fibroblasts) activation, initiating fibrogenesis (Mazzocca et al., 2005). TGFβ-1, which can initiate as a pro-fibrotic pathway, is upregulated in response to HCV replication and may contribute to collagen metabolism during HCV infection (Lin et al., 2010). Prolidase activity increases in HCV infection, and the combination of prolidase activity and oxidative damage leads to chronic viral hepatitis (Duygu et al., 2012). Liver fibrosis is due to the constant destruction of collagen in hepatic tissue and collagen accumulation in the ECM. Similar to HCV, patients with hepatitis B virus (HBV) infection have also been reported to display increased prolidase activity. HBV has a partially double-stranded DNA genome and belongs to the Hepadnaviridae family. However, in contrast to HCV, the authors found that HBV infection did not correlate with liver fibrosis (Duygu et al., 2013). A similar increase in prolidase activity was seen in pediatric HBV infection (Şen et al., 2014).

### Prolidase and Bone Diseases

The bone comprises of approximately 90% collagen (mostly type I collagen) (Clarke, 2008) PD patients exhibit symptoms such as a shortened stature, hypertelorism, and saddle nose. In addition, bone deformations were used to identify the first patient with PD (Seakins, 1963). Accordingly, PD mice are small with a short femur snout compared to healthy mice. This phenotype was prominent in mice at an early age (post-natal and pubertal), when collagen synthesis and degradation are the highest, demonstrating that prolidase is required for normal skeletogenesis (Besio et al., 2015). Serum prolidase activity is significantly lower in patients with benign joint hypermobility syndrome than in healthy individuals, similar to PD patients (Em et al., 2014).

Osteogenesis imperfecta (OI) is a genetic disorder characterized by defects in COL1A1 and COL1A2, affecting bone, skin, and other tissue with high collagen content. The mutated gene results in improper collagen metabolism (Nijhuis et al., 2019). OI and PD have similar phenotypes, and both have decreased prolidase activity. In OI fibroblasts, prolidase activity is diminished despite the amount of prolidase protein remaining the same. This, suggests a defect at the post-transcriptional and/or post-translation level, in contrast with PD patients wherein the effect is mainly genetic. Expression of beta-1 integrin and IGFR, which have been previously shown to regulate prolidase activity are decreased in OI (Galicka et al., 2001; Galicka et al., 2003). Decreased collagen synthesis is accompanied by decreased prolidase activity, β1 integrin, and IGFI receptors in OI fibroblasts (Galicka et al., 2005). Pharmacological therapy using type-1 bioactive compounds in OI treatment stimulate collagen biosynthesis by increasing prolidase activity and is accompanied by increased IGF-I receptor expression (Galicka and Nazaruk, 2007). Ankylosing spondylitis and Rheumatoid Arthritis have lower serum prolidase levels associated with decreased collagen turnover (Uçar et al., 2013). On the other hand, prolidase activity is increased in thalassemia major, which is associated with osteoporosis (Cakmak et al., 2010). There are no mechanisms reported so far for prolidase regulation in bone-associated diseases.

### Prolidase and Cancer

Prolidase activity has been found to be altered in a variety of cancers. An increase or decrease in prolidase activity can indicate the presence of a cancer and its progression. As a result, prolidase activity measurement can be a useful diagnostic marker for cancer detection and progression. Prolidase activity is elevated in several cancers, such as breast cancer, endometrial cancer, lung cancer, melanoma, and others (Karna et al., 2000; Cechowska-Pasko et al., 2006; Mittal et al., 2007; Arioz et al., 2009). The increase in prolidase activity in these cancers is due to increased collagen biosynthesis allowing for tumor metastasis. In most cancers, the benefit of early detection dramatically enhances the chance of post-diagnosis survival. Importantly, monitoring prolidase activity could be a way to evaluate the success of a treatment or cancer progression.

Collagen has been shown to regulate cellular growth and differentiation through interaction with integrin receptors and plays a vital role in tumorigenesis and invasiveness. The main factor contributing to collagen metabolism disturbances in cancer tissue is the deregulation of the balance between collagen biosynthesis and degradation. A decrease in tissue collagen content and β1-integrin receptor accompanied by an increase in prolidase activity was observed in breast cancer (infiltrating ductal carcinoma) tissue compared to normal tissue. The significantly higher prolidase activity levels indicated an increase in collagen turnover rate in tumor tissue than controls (Cechowska-Pasko et al., 2006). Another study found a correlation between elevated serum prolidase activity to the stage of the disease, tumor size, and prognosis of breast carcinoma (Bayhan et al., 2016). Enhancement of collagen turnover, accompanied by an increase in prolidase activity, was reported in stomach cancer (Guszczyn and Sobolewski, 2004). Prolidase activity was also significantly higher in patients with inoperable gastric cancer than in operable cases and the control group. There is also a strong correlation between tumor volume and prolidase activity (Celik et al., 2017). It has been reported that serum prolidase activity was significantly higher in patients with epithelial ovarian carcinomas (EOC) than healthy controls (Camuzcuoglu et al., 2009). The study observed that more advanced stages, higher tumor grades, and higher cancer antigen 125 (CA-125) levels among patients with EOC were associated with higher serum prolidase activity. Similarly, higher serum prolidase activity was observed in esophageal squamous cell carcinoma (ESCC) patients compared to healthy controls with a positive correlation between the stage of ESCC and prolidase activity (Sayır, 2019). In most cancers studied, aberrations in collagen metabolism were accompanied by significant differences in prolidase activity. Cumulatively, these studies suggest that enhanced prolidase activity in cancer tissues may contribute towards matrix degradation and remodeling of the tumor microenvironment towards a more invasive phenotype. As a result, it has been speculated that compounds that inhibit prolidase activity may have therapeutic potential as anticancer drugs.

### Prolidase and Respiratory Diseases

Idiopathic pulmonary fibrosis (IPF) is a chronic lung disease that results in fibrosis of the lungs (Barratt et al., 2018). Although the reasons are unknown, the pathobiology of IPF begins with fibroblastic foci (local accumulation of fibroblasts and myofibroblasts), and excessive deposition of collagen and ECM components, which result in the deformation of the lungs. Intraluminal fibrosis is a characteristic of IPF due to the recruitment of inflammatory cells, resulting in the synthesis and degradation of extracellular-associated components in the lung microenvironment (Oikonomidi et al., 2009; Barratt et al., 2018). Animal models of IPF show elevated prolidase activity that correlates with observed histopathology of lung fibrosis (Türkbeyler et al., 2012). IPF is associated with an increased risk of lung cancer in which collagen and prolidase activity also increase (Karna et al., 2000; Ballester et al., 2019). Unlike other cancers wherein beta-1 integrin levels were shown to be associated with prolidase regulation, there was no difference in β1 integrin levels in healthy lung cells versus cancerous cells, suggesting a different mechanism for prolidase regulation in lung cancer (Karna et al., 2000).

Asthma is characterized by inflamed and narrowed airways that produce excessive mucus, triggering breathing problems. Due to inflammation, increased collagen deposition narrows the airways (Oikonomidi et al., 2009). PD patients experience asthma-like manifestations, highlighting the crucial role of prolidase activity in lung health and pathologies. In blood sera collected from adults with bronchial asthma, prolidase activity was increased (Kaleli et al., 2006). However, another study found that serum prolidase activity decreased in children with bronchial asthma (Cakmak et al., 2009). These conflicting studies suggests the possibility of differential impact of age on prolidase activity. Some other collagenolytic enzymes are also associated with an age-related increase (Yu et al., 2013). There is no explanation why a decrease in prolidase activity, as in PD, causes the same pathology as an increase in prolidase activity in adults with bronchial asthma.

Pulmonary tuberculosis (PTB) is a severe bacterial infection of the lungs and one of the most common infectious diseases worldwide (Gumus et al., 2011). Serum prolidase activity was increased in moderate to severe stages of PTB. The increase in serum prolidase activity may be due to tissue destruction, increased immunoglobulin, increased complement levels, or increased fibroblastic activity, which occurs in PTB (Gumus et al., 2011; Wang et al., 2014). The same is seen in Tuberculous Pleurisy (Ipcioglu et al., 2008). Chronic obstructive pulmonary disease (COPD), another inflammatory lung disease that obstructs airflow in the lungs caused by smaller air passageways and irreversibly damaged alveoli, is marked by significantly lower plasma prolidase activity than healthy controls (Gencer et al., 2011).

Influenza is a highly infectious respiratory illness caused by either influenza A and influenza B viruses. Symptoms include sore throat, runny nose, cough, fever, headache, muscle pain, and fatigue (Krammer et al., 2018). Prolidase has been reported to function as a novel entry factor for influenza A virus. Prolidase knockdown and inhibition negatively affected influenza A virus fusion events and its colocalization with endosomal markers. Prolidase was shown to be essential for early endosomal routing upon viral entry (Pohl et al., 2014).

### Prolidase in Diabetes Mellitus (DM) and Kidney Disorders

Persistent hyperglycemia due to DM causes pronounced effects on the expression, organization, and modification of ECM components in many organs (Brownlee, 2001; Kolset et al., 2012). These complications lead to the development of diabetes-specific microvascular pathologies. Diabetic nephropathy (DN) is one of the major complication of long term DM and is the most common cause of end stage renal disease (ESRD). DN is associated with structural alterations in the glomerular basement membrane, a specialized ECM composed of type IV collagen, laminin, nidogen, and heparan sulfate proteoglycan, that connects podocytes and endothelial cells in the kidneys (Miner, 2012). In type II DN patients, the substantial damage to kidneys is associated with increased collagen accumulation due to increased thickening of the basement membrane. The development of kidney disease in these patients is marked by excretion of albumin into the urine and renal impairment. Serum prolidase activity is reported to be higher in type 2 DM patients with microalbuminuria than patients without microalbuminuria (Sabuncu et al., 2016). A previous study also reported that, DN patients with ESRD had higher serum prolidase activity, which positively correlated with glucose and creatinine levels (Verma et al., 2014). On the contrary, Erbagci et al. have reported decreased prolidase activity in patients with type II DM than in healthy volunteers with or without diabetic nephropathy (Erbaǧci et al., 2002). These studies thus suggest that the different stages of DN may have a variable effect on prolidase enzyme activity.

### Prolidase and Brain Diseases

The amino acid proline has been suggested to act as a neurotransmitter in brain, and high levels of proline have been associated with neurological symptoms and brain abnormalities (Wyse and Netto, 2011). Since proline is a product of prolidase catalytic activity, higher prolidase activity will increase proline levels. Proline that is not incorporated into collagen or other proteins is further sequentially catabolized to glutamate by proline oxidase/proline dehydrogenase and pyrroline-5-carboxylate dehydrogenase (Pandhare et al., 2009; Phang et al., 2010). A dysfunction or disorder in these enzymes can cause either type I or II hyperprolinemia (Wang et al., 2013; Plecko and Steinfeld, 2017). Hyperprolinemia is associated with cognitive deficits and intellectual disabilities and is associated with schizophrenia, Parkinson’s disease, Alzheimer’s disease, and cerebral ischemia. In addition, proline levels can affect glutamate levels in the brain, and an increase in prolidase may affect glutamate-proline dynamics. High activity of prolidase in the hippocampus and cerebellum regions has been reported in adult male rat brains (Chi et al., 2009). Rat brain prolidase also has a high affinity for Ser-Pro dipeptides. Serine activates a type of neurotransmitter receptor called the N-methyl-D-aspartate (NMDA) receptor (Van-Horn et al., 2013). The hippocampus is vital for connecting short-term memory to long-term memory and spatial memory to enable navigation (Squire et al., 2015). Damage or improper function of the hippocampus is associated with Alzheimer’s disease, stress, and post-traumatic disorder. The potentiation of glutamate excitotoxicity *via* NMDA activation has been suggested to be a possible mechanism for neurological dysfunction in hyperprolinemia (Cohen and Nadler, 1997; Delwing et al., 2003; Arikanoglu et al., 2013). Hyperstimulation by glutamate of NMDA receptors can lead to neuronal death. Elevated prolidase activity is thought to induce NMDA dysregulation by increasing plasma proline levels in schizophrenia (Bahceci et al., 2015; Güneş et al., 2016). T-Cell proteome of Parkinson’s disease patients treated with dopamine receptor antagonist showed increased levels of prolidase (Alberio et al., 2012). Similarly, in patients of Alzheimer’s disease and major depressive disorder, prolidase activity has been reported to be higher (Arikanoglu et al., 2013) (Kokacya et al., 2014). Recently, a study reported elevated prolidase activity in epileptic patients taking some antiepileptic drug treatment, suggesting an increased risk of subclinical vascular damage (Karacan et al., 2020). Patients with post-traumatic stress disorder have defects in the hippocampus and show decreased prolidase activity (Kitayama et al., 2005; Demir et al., 2016). Despite the lack of research demonstrating the direct impact of prolidase in these disorders, prolidase activity may serve as a useful marker in brain health and mental disorders, especially at different stages of the disease.

PD phenotype has been shown to include a moderate to severe degree of intellectual disabilities and developmental delays. High amounts of accumulating proline-containing peptides have been suggested as a possible explanation (Hui and Lajtha, 1980). Post-natal prolidase deficient model mice exhibit vascularization defects in the cerebral and cerebellar cortices linked to the role of prolidase in angiogenic signaling processes. Improper ECM remodeling causes meningeal defects affecting brain development resulting in impairments, loss of basement membrane integrity, and disorganization of the cytoarchitecture of the cerebral cortex (Insolia and Piccolini, 2014).

### Prolidase and Bacterial Infections

*Helicobacter pylori* (*H. pylori*) is a non-invasive spiral-shaped microaerophilic microorganism associated with severe gastric pathologies, such as peptic ulcers, chronic active gastritis, mucosal atrophy, and gastric adenocarcinoma. Upon infection, gastric atrophy occurs due to replacement of the gastric mucosal glands with collagen fibers. Persistent infection results in the architectural disruption of the ECM and fibrosis of the stomach’s mucosal layer. A study observed that *H. pylori*-positive subjects had higher serum prolidase activity than *H. pylori*-negative subjects (Aslan et al., 2007). Since this was an observational study, no mechanism was offered to explain why the serum prolidase activity may be higher. Though patients were selected based on health, other fibrotic events may contribute to the increased serum prolidase levels.

## Other Uses of Prolidase

Organophosphorus acid anhydrolases (OPAA) are enzymes that hydrolyze highly toxic compounds such as acetylcholinesterase-inhibiting compounds, chemical warfare G-type nerve agents, and pesticides. OPAA-2 from *Alteromonas* sp. strain JD6.5 EcoRI-lZAPII chromosomal library showed sequence homology with *E. coli* aminopeptidase P and human prolidase. OPAA-2 also demonstrates prolidase-like activity by cleaving leu-pro and ala-pro, suggesting an evolutionary relationship. Though OPAA performs this activity, it hydrolyzes P-F and P-O bonds while prolidase hydrolyzes C-N (Cheng et al., 1996). Further study suggested that *Alteromonas haloplanktis* OPAA is a type of prolidase. *Alteromonas* prolidase can hydrolyze G-type nerve agents and DFP, soman, sarin, and cyanide containing tabun, therefore is used as an antidote for poisoning (Cheng et al., 1997; Cheng et al., 1999; Petrikovics et al., 2000). Native and recombinant human prolidase also catalyzes these nerve agents with A252R or P365R substitution mutation of the enzyme, improving the catalytic activity against organophosphorus compounds (Chandrasekaran et al., 2013; Yun et al., 2017).

## Conclusion

Prolidase is an enzyme that catalyzes dipeptides containing a C-terminal proline or hydroxyproline. These dipeptides are produced primarily during collagen turnover, thus making prolidase mediated catalysis as the rate-limiting step in collagen biosynthesis. The generated pool of proline is recycled during collagen synthesis and turnover. This process is important in several physiological and pathological processes. Therefore, alterations in prolidase activity are associated with not only prolidase deficiency, a rare genetic disorder, but also with several pathological conditions. Prolidase activity is vital for wound healing, and prolidase deficiency is associated with poor wound healing in both mice and humans. Although prolidase is essential in various processes, fundamental research on the enzyme is limited. Thus, a better understanding of the regulation of prolidase both during normal and diseased conditions, will help identify novel pathways that can be exploited for possible therapeutic application, especially in patients suffering from chronic unhealing wounds.
